# Process Optimization for Ultra-Precision Machining of HUD Freeform Surface Mold Cores Based on Slow Tool Servo

**DOI:** 10.3390/mi17020164

**Published:** 2026-01-27

**Authors:** Tianji Xing, Naiming Qi, Huanming Gao, Longkun Xu, Xuesen Zhao, Tao Sun

**Affiliations:** 1 Center for Precision Engineering, Harbin Institute of Technology, Harbin 150001, China; xingtianji2020@foxmail.com (T.X.); xulongkun18@163.com (L.X.); zhaoxuesen@hit.edu.cn (X.Z.); 2 Department of Aerospace Engineering, Harbin Institute of Technology, Harbin 150001, China; qinmok@163.com; 3 Tianjin Navigation Instrument Research Institute, Tianjin 300131, China; wangbao_1903@126.com

**Keywords:** head-up display, optical freeform surface, single-point diamond turning, slow tool servo, ultra-precision manufacturing

## Abstract

With the rapid development of Head-Up Display (HUD) technology for vehicles, optical freeform mirrors, as its core optical components, are crucial for achieving system compactness and high imaging quality. However, their complex surface shapes and large-aperture characteristics pose significant challenges to ultra-precision manufacturing. This study presents a systematic optimization framework for the ultra-precision machining of HUD optical freeform mold cores, integrating surface design, tool path planning, vibration analysis, and process parameter optimization. Firstly, based on the XY polynomial freeform surface model, an off-axis three-mirror HUD system was designed, and the surface parameters and machining dimensions of the mold core were determined. For the Single-Point Diamond Turning (SPDT) Slow Tool Servo (STS) process, a hybrid trajectory planning method combining equidistant projection and cubic spline interpolation was proposed to ensure the smoothness and accuracy of the tool path. Through theoretical analysis and experimental verification, the selection criteria for tool parameters such as tool nose radius and effective cutting angle were clarified, and the mechanistic impact of *Z*-axis vibration on surface roughness and waviness was quantitatively revealed. Finally, through ultra-precision turning experiments and on-machine measurement, a high-precision freeform surface mold core was successfully fabricated. This validates the effectiveness and feasibility of the proposed process solution and provides technical support for the high-quality manufacturing of HUD optical elements.

## 1. Introduction

With the rapid development of intelligent driving technology and in-vehicle display systems, the Head-Up Display (HUD) has become a key optoelectronic device in modern automobiles for enhancing driving safety and convenience. Treat’s research indicates that by projecting driving information in the form of a virtual image into the driver’s forward field of view, superimposing it with the real road scene, HUDs can effectively avoid the blind spot caused by looking down at the instrument panel, thereby significantly reducing the risk of traffic accidents [1]. In recent years, Augmented Reality Head-Up Displays (AR-HUD) have become a research hotspot due to their more intuitive information presentation, involving aspects such as human–machine interface design [2], driver acceptance [3], and stereoscopic display technology [4].

As the core component of the HUD optical system, optical freeform mirrors exhibit irreplaceable advantages in achieving system compactness and improving imaging quality due to their complex surface characteristics, such as asymmetry and large off-axis angles. Some scholars have conducted a series of studies and summaries in this direction. Kumar et al. reviewed progress in the design and manufacturing of novel freeform optics, highlighting its application value in multiple cutting-edge fields [5]. Optical freeform surfaces refer to complex optical surfaces without a unified axis of rotational symmetry. They are typically described by discrete point clouds or parametric equations. Yang et al. proposed a point-by-point three-dimensional construction-iteration method for the direct design of freeform surfaces and imaging systems, and also systematically analyzed the aberrations of freeform terms on decentered and tilted optical surfaces [6], providing theoretical support for the application of freeform surfaces in asymmetric systems [7]. Compared to traditional spherical or aspheric optical elements, freeform surfaces offer higher degrees of freedom in optical design, enabling effective aberration correction and optical path optimization, and have been successfully applied in the design of automotive HUD systems based on freeform reflection [8]. Feng et al. developed a simplified freeform optical design method for complex laser beam shaping, demonstrating its powerful capability in light field manipulation [9]. Consequently, freeform optical technology has found wide application in various high-end fields such as aerospace, medical imaging, and laser processing. However, the fabrication of such large-aperture freeform surfaces poses significant challenges in terms of tool path planning, vibration control, and surface integrity, particularly when using ultra-precision diamond turning.

The complex surface characteristics of freeform surfaces also pose significant challenges to their ultra-precision manufacturing. This is particularly true for the reflector mold cores used in HUDs, which typically have large apertures of 100–300 mm, millimeter-level sagittal height variations, and high surface form accuracy requirements. Their machining quality directly determines the imaging performance of the final optical system. Currently, ultra-precision machining technologies suitable for manufacturing optical freeform surfaces mainly include Single-Point Diamond Turning (SPDT), ultra-precision grinding, lapping and polishing, and post-processing techniques for additive manufacturing [10]. Among these, SPDT technology has become the mainstream process for manufacturing freeform optical components due to its nanometer-level machining accuracy, excellent surface quality, and high machining efficiency. Zhang et al. utilized iterative learning control to achieve model-free tool path modification in freeform surface ultra-precision turning, demonstrating the role of advanced control algorithms in improving machining accuracy [11]. Nguyen et al. applied a Bayesian optimization machine learning model to predict surface roughness in the SPDT of polycarbonate, reflecting the application of intelligent technology in process optimization [12]. Nagayama et al. proposed a deterministic error compensation method for freeform surfaces in slow tool servo (STS) diamond turning to achieve nanometer-level form accuracy [13]. Sato et al. developed tool path generation and optimization methods for freeform surface diamond turning based on independently controlled fast tool servos [14].

Within SPDT technology, STS and Fast Tool Servo (FTS) are two typical methods for machining complex surfaces. Gu et al. investigated the influence of system dynamics on the surface topography of microlens arrays in fast tool servo-based diamond turning, revealing the importance of dynamic factors in micro-machining [15]. FTS achieves high-frequency micro-displacement through an independently driven fast servo tool holder, making it suitable for machining components with small *Z*-axis strokes, such as micro-structure arrays. Its tool path generation methods have also been developed for multi-degree-of-freedom systems [16]. In contrast, STS technology relies on the coordinated motion of the X, Z, and C axes, enabling continuous trajectory control with large strokes and high stiffness. It is particularly suitable for the efficient machining of large-scale freeform optical elements [17]. An et al. manufactured a freeform reflector for an HUD system using STS technology and completed its measurement, validating the feasibility of STS for machining large-scale freeform surfaces [18]. STS technology can also be combined with ultrasonic vibration [19] or diamond wheel grinding [20,21] to further expand its process capabilities, making it especially suitable for machining optical elements like HUD freeform surfaces, which feature gently varying surface shapes and large apertures.

Simultaneously, scholars have conducted extensive research on HUD freeform surface design and machining. Lee et al. [22] designed an off-axis three-mirror optical path based on Zernike polynomial freeform surfaces, achieving a more compact folded optical path and aberration suppression. Research on ultra-precision machining of freeform mold cores for advanced HUD equipment has also confirmed the feasibility of this technology [23]. Park et al. [24] successfully achieved ultra-precision turning of an HUD mold core using a Moore Nanotech ultra-precision lathe and STS technology, obtaining a machined surface with a PV value below 1 μm and an Ra below 20 nm.

Regarding the inspection of optical freeform surfaces, commonly used measurement methods include optical interferometry, structured light scanning, and coordinate measurement. Zygo’s (Middlefield, CT, USA) white light interferometers can achieve sub-nanometer roughness measurement, while laser phase-shifting interferometers (such as the Verifire™ series) are suitable for nanometer-level detection of surface form errors and can utilize methods like convolutional neural networks to enhance calibration capabilities for freeform surface alignment errors [25]. Structured light technology is another important category. Xing et al. [26] proposed a high-precision online calibration method for structured light 3D measurement, improving the accuracy of optical measurement. The Institute of Optics and Electronics, Chinese Academy of Sciences, successfully measured the surface shape of an HUD reflector using structured light technology, with results consistent with those from an interferometer. For complex surfaces, advanced methods such as phase retrieval [27], computer-generated holography [28], and robot-assisted dynamic path planning measurement [29] have also been developed. Wang et al. [30] investigated the generation and material removal mechanism of double-conical optical surfaces based on STS and diamond wheel grinding, providing a process foundation for on-machine inspection. Profilometers from Taylor Hobson [31], as representatives of mechanical stylus-based measurement, are still widely used today for surface topography detection, while multi-source data fusion methods at the micro-nano scale aim to address the challenge of cross-scale measurement integration [32].

In summary, the ultra-precision machining of automotive HUD optical freeform mold cores is a comprehensive technology involving optical design, tool path planning, process parameter optimization, and precision inspection. Factors during the machining process, such as vibration [33], material removal mechanisms [34], and diamond tool wear [35], are all key factors affecting the final quality. Currently, this field still faces challenges, including dependence on imported high-end equipment, incomplete process databases, and a lack of independent software. Therefore, this study not only addresses the practical manufacturing challenges of HUD freeform molds but also contributes systematically to the science of ultra-precision machining. Key advancements include the development of a hybrid tool path planning strategy for large-aperture rectangular freeform surfaces, the establishment of a vibration diagnostic framework linking *Z*-axis vibration characteristics to surface waviness patterns, and the implementation of a closed-loop manufacturing workflow integrating in-process measurement with adaptive error compensation. These contributions enhance the fundamental understanding of precision machining mechanisms for complex optical surfaces and provide a valuable reference for further theoretical and technological development in the field.

This study focuses on the single-point diamond STS turning of HUD optical freeform mold cores, systematically investigating surface parameter optimization, tool trajectory planning, analysis of factors influencing surface quality, and process parameter optimization. The aim is to establish an efficient and high-precision machining process solution, providing technical support for the independent manufacturing of HUD optical components.

## 2. Design Methodology

### 2.1. HUD Freeform Surface Parameter Design

**Figure 1:**
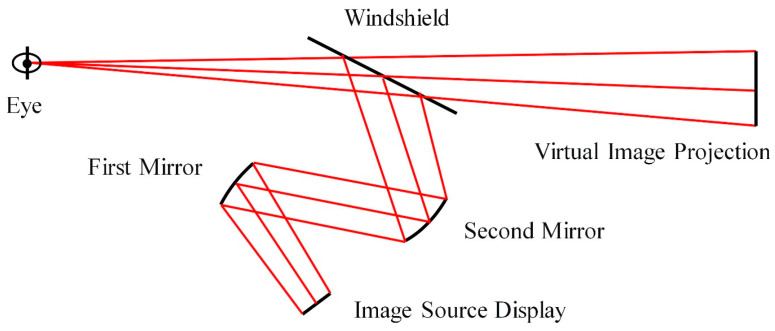
Schematic diagram of the HUD optical system.

The optical system of an automotive HUD typically adopts an off-axis three-mirror configuration. Its optical path consists of three main modules: the image source display, a freeform mirror group, and the windshield, as illustrated in Figure 1. The light emitted from the image source is first folded by the primary mirror, then magnified and corrected for aberrations by the secondary mirror, and finally forms a virtual image through the windshield. To achieve a large field-of-view, low-distortion virtual image projection within a confined space, the surface design and optimization of the freeform mirror are crucial.

In an off-axis three-mirror optical system, utilizing an optical freeform surface as the reflector not only eliminates complex aberrations but also significantly enhances overall system performance. Polynomial freeform surfaces are among the most common construction equations for HUD reflectors. Its mathematical representation is based on a conic surface augmented with a series of associated polynomials. The general mathematical expression is

(1) $$z=\frac{cr^{2}}{1+\sqrt{1-\left(1+k\right)c^{2}r^{2}}}+\sum_{i=1}^{N}C_{i}P_{i}(\rho ,\varphi )$$

where *c* is the surface curvature, *k* is the conic constant, *C_i_* are the polynomial coefficients, *r* is the radial coordinate, *N* is the total number of polynomial terms, and *P_i_* represents the *i*-th polynomial basis function with *ρ* being the normalized radial coordinate and *φ* the azimuthal angle. This model offers advantages such as high design freedom, strong aberration correction capability, and ease of data exchange. Common polynomial sets for *P_i_* include Chebyshev polynomials [36], Q-type polynomials [37], Zernike polynomials [38], and XY polynomials [39], among others.

Due to its high design freedom, strong aberration correction capability, and ease of data exchange with machining equipment, the XY polynomial freeform surface is particularly suitable for designing HUD optical freeform mirrors. Therefore, this study employs the XY polynomial freeform surface to characterize the windshield and workpiece surface shapes. Its specific expression is

(2) $$z=\frac{cr^{2}}{1+\sqrt{1-\left(1+k\right)c^{2}r^{2}}}+\sum_{i=1}^{N}C_{i}x^{m}y^{n}$$

In this expression, *m* and *n* denote the exponents of *x* and *y*. The automobile front windshield is a crucial component. As its surface shape is an irregular curve, it also constitutes a type of freeform surface. To obtain the mathematical equation for the windshield surface for subsequent optical modeling, discrete point cloud data for a specific car model’s front glass was acquired from a windshield manufacturer. This study converted these point clouds into an XY polynomial freeform surface representation. The specific parameters are listed in Table 1, corresponding to a 3rd-order polynomial equation:

(3) $$z=\frac{cr^{2}}{1+\sqrt{1-\left(1+k\right)c^{2}r^{2}}}+C_{1}+C_{2}x+C_{3}y+C_{4}x^{2}+C_{5}xy+C_{6}y^{2}+C_{7}x^{3}+\cdots$$

**Figure 2:**
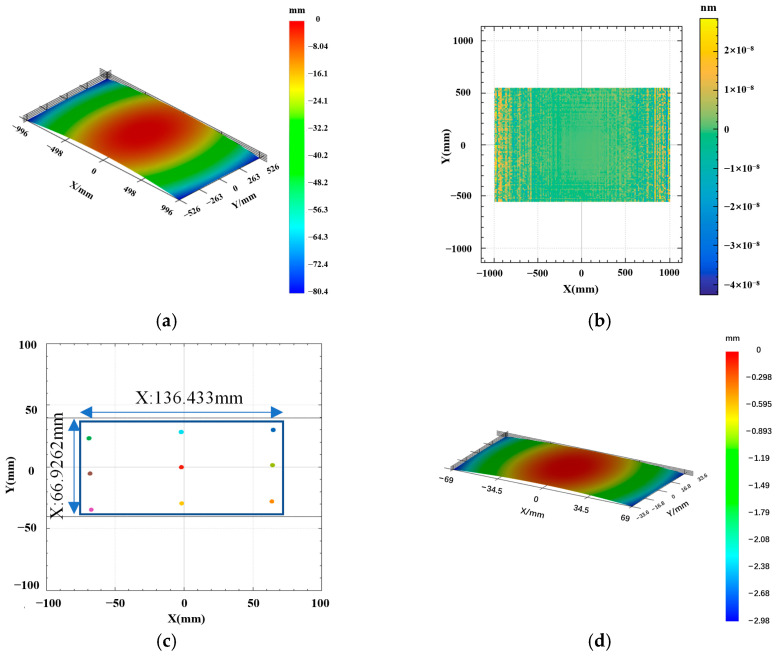
Fitted windshield surface and corresponding error: (**a**) fitted windshield surface; (**b**) fitting error of the windshield; (**c**) HUD ray tracing dimensions; (**d**) 3D rendering of the HUD optical freeform surface.

The 3D rendered image of the fitted windshield is shown in Figure 2. Figure 2a displays the converted fitted windshield surface, while Figure 2b shows the fitting error of the freeform surface equation. The maximum error is found to be less than 8 × 10^−8^ nm, indicating a good fit.

This study designed an HUD imaging optical path system with a virtual image distance of 2.5 m. Through analysis and optimization, the required surface parameters for the optical mirror were derived. The basic performance parameters for constructing the HUD optical system include virtual image distance, field of view, eyebox, and down-angle. Based on the analysis of virtual image parameters of commercially available HUD systems, the optical system parameters were determined as shown in Table 2.

The dimensions of the HUD optical freeform surface designed according to the optical system parameters are shown in Figure 2c,d. The actual footprint of the light rays on the reflector is 136.433 mm × 66.9262 mm. Therefore, the machining dimensions were determined to be 138 mm × 68 mm. The sagittal height along the *Z*-axis is approximately 2.98 mm, making it suitable for ultra-precision machining via Single-Point Diamond STS turning.

The surface shapes of the HUD optical freeform mirror and its mold core are complementary. Consequently, the surface shape of the mold core corresponding to the optimized optical freeform mirror can be expressed using the XY polynomial freeform surface equation parameters, as shown in Equation (3). The specific data are listed in Table 3.

### 2.2. Single-Point Diamond Turning Tool Selection

Monocrystalline diamond possesses exceptional properties such as extreme hardness, excellent wear resistance, high strength, good thermal conductivity, low thermal expansion coefficient, and a sharp cutting edge. Consequently, it is commonly fabricated into cutting tools for ultra-precision machining. When machining freeform surfaces, due to their complex shapes, it is crucial to select appropriate tool parameters to avoid interference during the turning process and ensure the tool tip can properly engage with the workpiece. Key parameters include the tool nose radius (*R_t_*), effective cutting angle (*β*), rake angle (*γ*), and clearance angle (*α*).

**Figure 3:**
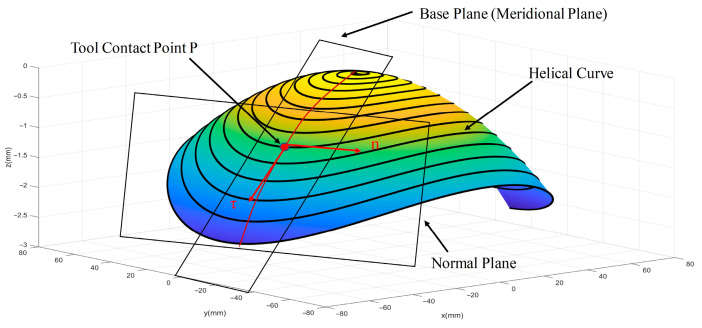
Diagram of the parameter relationship between the tool and the workpiece.

To facilitate the description of surface slopes and curvatures in subsequent sections, the radial cross-section of the workpiece is defined as the base plane, and the plane perpendicular to it is defined as the normal plane. Since the machined workpiece is non-rotationally symmetric, the tool tip moves along a helical path around the *Z*-axis. The slope of this helical curve continuously changes, and the spacing between adjacent turns is very small, making the tool path approximately circular. Therefore, the slope of this helical curve is termed the “sagittal slope,” and its curvature the “sagittal curvature.” The radial cross-section coincides with the meridional plane; hence, the corresponding curve slope and curvature are termed the “meridional slope” and “meridional curvature, respectively, as illustrated in Figure 3.

The selection of the tool nose radius *R_t_* involves considering any radial cross-section of the freeform workpiece surface to obtain a section curve. For the concave portions of this curve (convex portions do not constrain the tool nose radius), the maximum curvature—i.e., the maximum meridional curvature—is calculated. After determining the maximum meridional curvature from all radial cross-sectional curves, the selected tool nose radius must be smaller than the corresponding minimum radius of curvature [40]. The specific calculation is shown in Equation (4).

(4) $$R_{C}=\min\left\{\frac{{\left[1+{\left(\frac{\partial f(\rho ,\theta )}{\partial \rho }\right)}^{2}\right]}^{\frac{3}{2}}}{\left|\frac{\partial^{2}f(\rho ,\theta )}{\partial \rho^{2}}\right|}\right\}\;\;\;\;\;0<\rho <R$$

**Figure 4:**
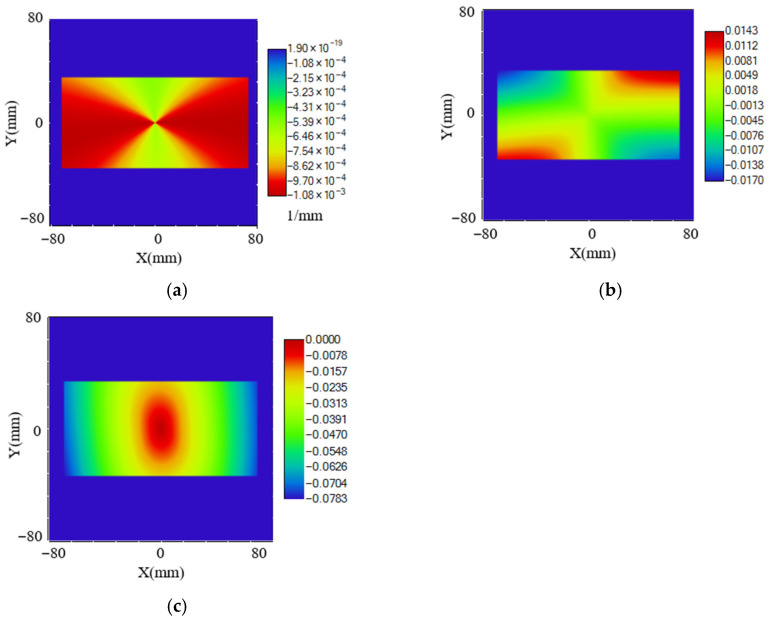
Variation in the meridional direction on the workpiece surface: (**a**) Meridional curvature of the workpiece surface. (**b**) Meridional slope of the workpiece surface. (**c**) Sagittal slope of the workpiece surface.

The meridional curvature of the workpiece surface is shown in Figure 4. All directions along the base plane are convex surfaces. Therefore, regarding interference avoidance between the tool and workpiece, the tool nose radius is not constrained in this aspect.

However, concerning tool nose radius compensation, particularly for unidirectional tool normal radius compensation, constraints on the tool radius are also required to ensure the approximation error remains within a specified range. As shown in Figure 4, the meridional curvature and meridional slope of the surface are correlated. Therefore, the meridional curvature and slope at their maximum point are used to calculate the maximum approximation error, thereby determining the permissible tool nose radius. Setting the maximum permissible error *E_P_* to 5 nm and substituting the maximum meridional curvature and slope values into Equation (5) yields that the maximum permissible tool nose radius must not exceed 1229.74 μm.

(5) $$R_{t}=\frac{E_{P}+\sqrt{{E_{P}}^{2}+K^{2}(2RE_{P}+{E_{P}}^{2})}}{K^{2}}$$

For the selection of the effective cutting angle *β* (tool arc included angle, tool nose arc enveloping angle), the tool tip moves along the helical curve equation. As shown in Figure 4a, on its radial cross-section, let the maximum absolute value of the meridional slope at the tool contact point P be KP. To avoid the need for tool offsetting and simplify tool mounting, the condition tan*β* ≥ 2*K_P_*tan*β* ≥ 2*K_P_* must be satisfied, i.e.,

(6) $$\beta \geq \arctan2\;K_{P}$$

From Figure 4b, *K_P_*_max_ = 0.0783. Substituting this into Equation (6) yields *β_c_* = 8.9002°. However, a larger effective cutting angle is not always better. A larger included angle results in a longer arc length of the tool nose, potentially reducing precision at the arc ends. A typical selection is 120°.

The selection of the rake angle *γ* and clearance angle αα depends on the cutting direction. The rake angle is the angle between the rake face and the tool base plane, and the clearance angle is the angle between the flank face and the cutting plane. Generally, the minimum non-interfering rake and clearance angles are determined based on the magnitude of the sagittal slope. During turning, *γ_c_* depends on the slope along the cutting direction, while *α_c_* depends on the slope opposite to the cutting direction. When the machining direction is uncertain, the maximum absolute slope *K_P_* can be used:

(7) $$\left\{\begin{array}{c} \gamma_{C}=\min({\mathrm{arccotK}}_{P})\\ \alpha_{C}=\max({\mathrm{arctanK}}_{P}) \end{array}\right.$$

From Figure 4c, taking *K_P_* = 0.017 and substituting into Equation (7) yields *γ_C_* = 89.0261° and *α_C_* = 0.9739°.

Beyond satisfying non-interference conditions, the selection of rake and clearance angles must also consider factors such as cutting temperature and workpiece material. For non-ferrous metals (e.g., copper, aluminum), the maximum negative rake angle generally should not exceed 10°. If no special requirements exist, a rake angle of 0° is typically used. The primary function of the clearance angle is to reduce friction between the tool flank face and the workpiece surface. A larger clearance angle reduces this friction, which is beneficial for improving surface quality. However, an excessively large clearance angle weakens the cutting edge, adversely affecting tool life. Therefore, a clearance angle of 10° is generally chosen.

### 2.3. Tool Path Point Generation Method

The core of machining optical freeform surfaces via single-point diamond ultra-precision STS turning lies in generating smooth tool paths while meeting the residual height accuracy requirement. Helical trajectories are the preferred choice due to their continuity, smoothness, and excellent alignment with the motion principles of the machining platform.

In turning trajectory planning, both the constant curve spacing method and the constant projection spacing method aim to maintain consistent spacing between adjacent tool paths. The constant curve spacing method requires equal spatial distances between curves. However, on curved surfaces, variations in surface curvature can lead to changes in the actual spacing, causing fluctuations in machine feed rates. This imposes higher demands on motion control and may negatively impact surface quality. In contrast, the constant projection spacing method ensures a constant distance between tool path projections onto the X-Y plane, independent of surface curvature. This method offers simpler calculations, stronger adaptability, effectively simplifies CNC programming, reduces control difficulty, and thereby enhances machining efficiency and quality. Therefore, this study employs the constant projection spacing method.

**Figure 5:**
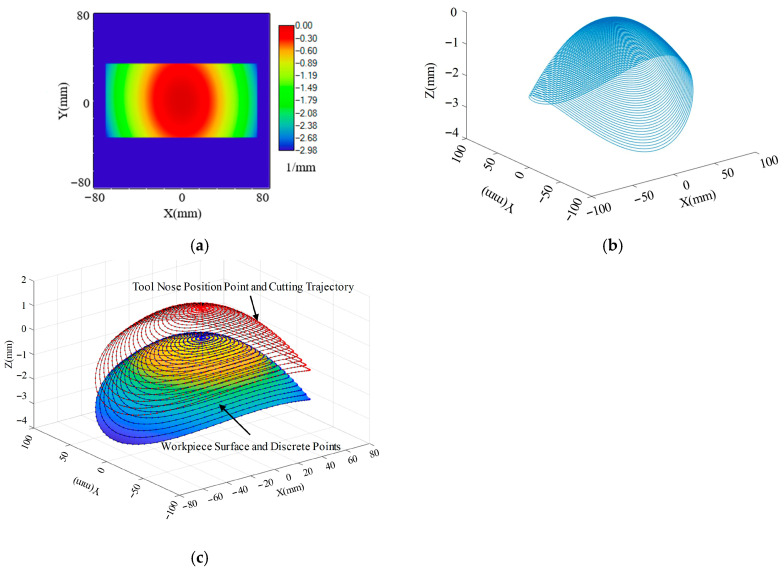
HUD mold rendering and tool trajectory: (**a**) 3D rendering of the HUD mold. (**b**) Tool trajectory on the HUD mold surface. (**c**) Schematic of trajectory planning.

In this study, the optical freeform surface to be machined projects a rectangular contour onto the X-Y plane, as shown in Figure 5a. This shape is more suitable for the characteristics of the human visual system and the information presentation needs of drivers. Rectangular shapes are easier for the human eye to accept and process, and they can display more information, such as speed, navigation prompts, and warning messages, within a limited space. Additionally, rectangular shapes are easier to integrate with equipment like dashboards and windshields. Therefore, for such non-circular machined surfaces in actual STS turning, there will be sections of the tool path where the tool does not contact the workpiece, known as the “air-cut” or non-cutting segments [41]. Although the air-cut segments do not involve actual material removal, the tool motion trajectory in these sections directly influences the dynamic performance of the machine tool, subsequently affecting the surface quality of the workpiece.

Research on tool path generation for the air-cut segments can be approached from two directions. The first involves interpolating and completing the tool path for the air-cut section based on a known partial tool path curve. The second approach involves extending the existing surface shape through surface interpolation to obtain a surface whose projection onto the X-Y plane is circular. Then, the complete tool path, including the air-cut segments, can be calculated from this extended surface shape.

In this study, the surface equation of the HUD reflector mold core planned for machining is

(8) $$z=f\left(x,y\right),-69<x<69,-34<y<34$$

Since the three-axis ultra-precision machine tool machines the surface through coordinated motion of the X, Z, and C axes, it is necessary to convert the Cartesian coordinate mathematical equation of the freeform surface into a polar coordinate representation. Given the Cartesian equation of the reflector mold core surface, let *ρ* be the distance from any point *P* on the cutting trajectory to the coordinate origin in the X-Y projection plane and *θ* be the angle rotated relative to the starting point. Substituting *x* = *ρ*cos*θ* and *y* = *ρ*sin*θ* into the coordinate system yields the polar coordinate surface equation of the optical freeform surface:

(9) $$z=f(\rho ,\theta )=\frac{c\rho^{2}}{1+\sqrt{1-\left(1+k\right)c^{2}\rho^{2}}}+C_{1}+C_{2}\rho \cos\theta +C_{3}\rho \sin\theta +C_{4}\rho^{2}\cos^{2}\theta +\cdots$$

Software simulation shows that the helical tool path generated by extending the original freeform surface equation appears smooth, as illustrated in Figure 5b.

In the turning of optical freeform surfaces, trajectory interpolation is required after tool nose radius compensation. Traditional linear and circular arc interpolation can easily induce vibrations. High-order polynomial interpolation provides smoothness but may suffer from divergence. Piecewise, low-order polynomials offer better convergence but lack sufficient smoothness. Spline interpolation combines the advantages of both. Among spline methods, the cubic spline interpolation with specified second derivatives (using bending moments) achieves high machining accuracy by controlling curvature, while the cubic spline interpolation with specified first derivatives (using turning angles) is more suitable for curved trajectories. Considering the requirements for surface accuracy and smoothness comprehensively, this study employs the cubic spline interpolation method with specified second derivatives (three-bending-moment method) for trajectory interpolation.

Let the piecewise expression of the cubic spline interpolation be *s*(*t*). Since *s*(*t*) is a piecewise cubic polynomial that is second-order smooth (*C*^2^ continuous), its second derivative *s*″(*t*) is a piecewise linear continuous function, which can be expressed as

(10) $$s^{\prime \prime }\left(t\right)=\frac{t_{j+1}-t}{h_{j+1}}M_{j}+\frac{t-t_{j}}{h_{j+1}}M_{j+1},\;\;\;\;\;t\in \left[t_{j},\;t_{j+1}\right]$$

where *M_j_* are parameters to be determined; *h_j_*_+1_ = *t_j_*_+1_ − *t_j_* is the time difference between adjacent points, for *j* = 0, 1, 2, …, *n*−1.

Integrating *s*″(*t*) twice over this interval yields the expressions for *s*′(*t*) and *s*(*t*):

(11) $$s^{\prime }\left(t\right)=-\frac{{\left(t_{j+1}-t\right)}^{2}}{2h_{j+1}}M_{j}+\frac{{\left(t-t_{j}\right)}^{2}}{2h_{j+1}}M_{j+1}+A_{j},\;\;\;\;\;t\in \left[t_{j},\;t_{j+1}\right]$$

(12) $$s\left(t\right)=\frac{{\left(t_{j+1}-t\right)}^{3}}{6h_{j+1}}M_{j}+\frac{{\left(t-t_{j}\right)}^{3}}{6h_{j+1}}M_{j+1}+A_{j}\left(t-t_{j}\right)+B_{j},\;\;\;\;\;t\in \left[t_{j},\;t_{j+1}\right]$$

where *A_j_* and *B_j_* are constants of integration.

Using the interpolation conditions *s*(*t_j_*) = *f*(*t_j_*) and *s*(*t_j_*_+1_) = *f*(*t_j_*_+1_), the following equations can be solved simultaneously:

(13) $$\left\{\begin{array}{c} A_{j}=\frac{f\left(t_{j+1}\right)-f\left(t_{j}\right)}{h_{j+1}}-\frac{h_{j+1}}{6}\left(M_{j+1}-M_{j}\right)\\ B_{j}=f\left(t_{j}\right)-\frac{{h_{j+1}}^{2}}{6}M_{j} \end{array}\right.$$

Since *s*′(*t*) is continuous at the nodes, *s*′(*t_j_*_+0_) = *s*′(*t_j_*_−0_). Combining Equations (11) and (13) yields

(14) $$\frac{h_{j}}{6}M_{j-1}+\frac{h_{j}+h_{j+1}}{3}M_{j}+\frac{h_{j+1}}{6}M_{j+1}=\frac{f\left(t_{j+1}\right)-f\left(t_{j}\right)}{h_{j+1}}-\frac{f\left(t_{j}\right)-f\left(t_{j-1}\right)}{h_{j}}$$

(15) $$\mu_{j}M_{j-1}+2M_{j}+\lambda_{j}M_{j+1}=6f\left[t_{j-1}t_{j}t_{j+1}\right]$$

where *f*[*t*_*j*−1_,*t_j_*,*t*_*j*+1_] is the second-order divided difference of *f*(*t*) at *t_j_*_−1_, *t_j_*, *t_j_*_+1_.

Equation (15) contains n + 1 unknown parameters *M_j_*, but only provides *n* − 1 equations. Therefore, boundary conditions must be introduced. Using the first-type (clamped) boundary conditions (*s*′(*t*_0_) = 0, s′(*t_n_*) = 0), the following linear system of equations is obtained:

(16) $$\left(\begin{array}{cccccc} 2 & 1 & & & & \\ \mu_{1} & 2 & \lambda_{1} & & & \\ & \mu_{2} & 2 & \lambda_{2} & & \\ & & \ddots & \ddots & \ddots & \\ & & & \mu_{n-1} & 2 & \lambda_{n-1}\\ & & & & 1 & 2 \end{array}\right)\left[\begin{array}{c} M_{0}\\ M_{1}\\ M_{2}\\ \vdots \\ M_{n-1}\\ M_{n} \end{array}\right]=\left[\begin{array}{c} d_{0}\\ d_{1}\\ d_{2}\\ \vdots \\ d_{n-1}\\ d_{n} \end{array}\right]$$

It is readily verifiable that the coefficient matrix of this system is strictly diagonally dominant, guaranteeing a unique solution. This linear system can be solved efficiently using the Chasing Method (Thomas algorithm). The solved *M_j_* values are then substituted into Equation (12) to obtain the piecewise cubic spline interpolation expression *s*(*t*), completing the interpolation process. A schematic diagram is shown in Figure 5c, illustrating the final turning trajectory.

## 3. Analysis and Optimization of Influencing Factors

Numerous and intricate factors influence workpiece surface quality. This study focuses on analyzing the impact of machining trajectory, process parameters, and vibration on surface quality.

### 3.1. Tool Path Smoothness and Arc Length Analysis

The quality of the cutting trajectory is a direct factor affecting surface form accuracy. The ideal cutting trajectory is a smooth, continuous curve, and the resulting surface is termed the nominal surface. In actual machine tool machining, since the machine interprets a series of discrete points, the cutting path, even after interpolation, comprises a series of line segments between points. Taking the trajectory between two adjacent cutting points, let the constant arc length increment be Δ*l*, the local inclination angle be φ, and the arc with the maximum curvature of the nominal path in this segment be considered the ideal trajectory. The maximum linear error of the cutting trajectory is bounded by *E_Z_* ≤ Δ*Z.* Given a specified maximum error ΔZ, the corresponding allowable arc length increment can be determined from geometric relationships:

(17) $$h=\frac{1}{\kappa }-\sqrt{\frac{1}{\kappa^{2}}-\frac{\Delta l^{2}}{4}}$$

(18) $$\Delta Z=h\sqrt{K^{2}+1}$$

where *κ* is the sagittal curvature, and *K* is the sagittal slope of the surface.

Combining Equations (17) and (18) yields

(19) $$\Delta l=\sqrt{\frac{8\Delta Z^{2}}{\kappa \sqrt{1+K^{2}}}-\frac{4\Delta Z^{2}}{1+K^{2}}}$$

Based on the target form error (Δ*Z* ≤ 0.1 μm), the maximum allowable arc length increment is calculated as 272.15 μm. Therefore, Δ*l* = 0.27 mm was selected.

The radius at the transition between constant-angle and constant-arc-length zones is given by

(20) $$\rho =\frac{m\Delta l}{2\pi }$$

where *m* is the number of cutting points per revolution in the constant-angle zone. With *m* = 720 and Δ*l* = 0.27 mm, *ρ* = 30.96 mm.

### 3.2. Process Parameter Analysis

Beyond form accuracy, surface roughness is another critical parameter for evaluating optical surface quality. It is primarily determined by the cutting residual height, which is influenced by cutting parameters. Let the feed rate be *F* (μm/min), the spindle speed be *n* (rpm), and the feed per revolution be *f* (μm/rev). Their relationship is

(21) f=F/n

During freeform surface turning, a residual height is generated. For the studied convex surface with gently varying meridional curvature, the curve segment between adjacent points at a constant angle can be approximated as a straight line. Let the angle between this line and the horizontal be *β* (0 ≤ *β* ≤ 90°), and the distance between points be *L*. From geometry,

(22) $$L=\frac{f}{\cos\beta }$$

The theoretical residual height on the workpiece surface is therefore

(23) $$\varepsilon_{t}=R_{t}-\sqrt{{R_{t}}^{2}-\frac{L^{2}}{4}}=R_{t}-\sqrt{{R_{t}}^{2}-\frac{f^{2}}{4\cos^{2}\beta }}$$

The maximum residual height occurs at *β* = 0°. To ensure *ε_t_* remains below 5 nm, the feed per revolution and feed rate must satisfy

(24) $$\left\{\begin{array}{c} f\leq 2\sqrt{2R_{t}\varepsilon_{t}-{\varepsilon_{t}}^{2}}\\ F\leq 2n\sqrt{2R_{t}\varepsilon_{t}-{\varepsilon_{t}}^{2}} \end{array}\right.$$

**Figure 6:**
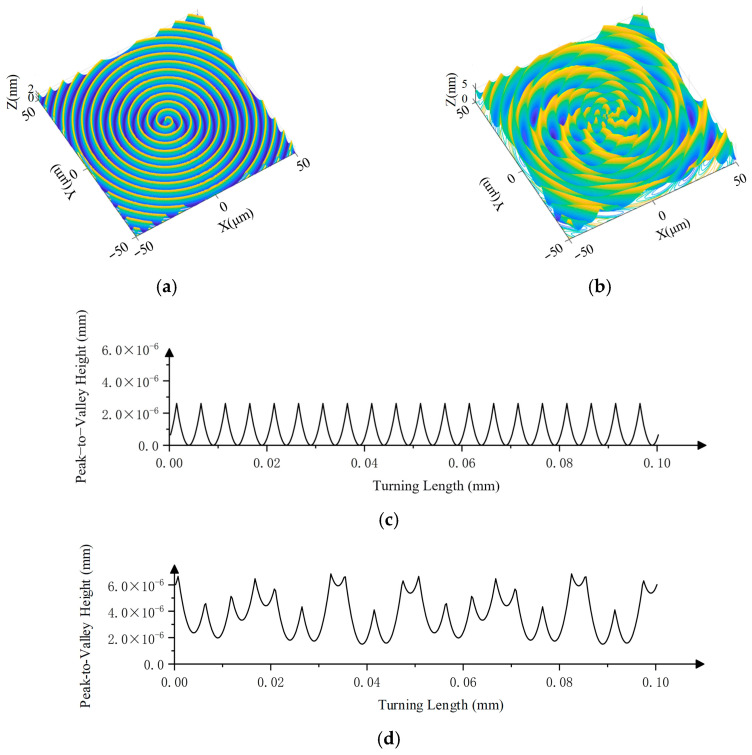
Simulated ideal turning surface: (**a**) 3D simulation of ideal turning. (**b**) 3D simulation of turning with vibration. (**c**) 2D profile of ideal turning. (**d**) 2D profile of turning with vibration.

Given the constraint, *f* ≤ 6.928 μm/rev. Using the simulation parameters listed in Table 4, the simulated surface micro-topography under ideal conditions is shown in Figure 6a,c.

### 3.3. Vibration Impact Analysis

**Figure 7:**
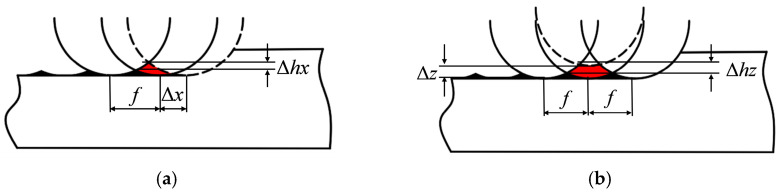
Schematic of tool–workpiece vibration effects: (**a**) *X*-axis vibration; (**b**) *Z*-axis vibration.

The above roughness analysis is based on ideal conditions, depending only on the tool nose radius and feed. In practice, factors such as axis coordination and stress variations induce vibration between the tool and workpiece, causing misalignment [42,43]. Vibration can be decomposed into *X*-axis and *Z*-axis components (Figure 7). The solid black arc represents the ideal tool position, while the dashed line shows the deviation due to vibration.

Given the ideal feed per revolution f and tool nose radius *R_t_*, the nominal residual height is *ε_t_*. Let vibration amplitudes be Δ*x* and Δ*z*, causing additional residual heights Δ*h_x_* and Δ*h_z_* relative to the ideal. From geometry,

(25) $$\Delta h_{x}=\sqrt{{R_{t}}^{2}-\frac{f^{2}}{4}}-\sqrt{{R_{t}}^{2}-\frac{{\left(f+\Delta x\right)}^{2}}{4}}$$

(26) $$\Delta h_{z}=\sqrt{{R_{t}}^{2}-\frac{f^{2}}{4}}-R_{t}\sin\left(\arccos\frac{\sqrt{f^{2}+\Delta z^{2}}}{2R_{t}}-\arcsin\frac{\Delta z}{\sqrt{f^{2}+\Delta z^{2}}}\right)$$

In ultra-precision machining, tool-workpiece vibration is in the nanometer range. For Δ*x* = Δ*z* = 5 nm, *f* = 5 μm, *R_t_* = 1.2 mm, calculations yield Δ*h_x_* = 5.211 × 10^−3^ nm and Δ*h_z_* = 3.1 nm. The effect of *X*-axis vibration is negligible; therefore, only *Z*-axis vibration is considered.

A simplified model assuming homogeneous, isotropic material, ideal chip removal, and a stable environment was used. Vibration was modeled as a sum of cosine functions:

(27) $$V\left(t\right)=\sum A_{Vi}\cos\left(2\pi f_{Vi}t+\phi_{i}\right)$$

where *A_Vi_*, *f_Vi_*, and *φ_i_* are the amplitude, frequency, and phase of each component, respectively.

The spindle rotational period is *T_n_* = 60/n seconds, with frequency *f_n_* = n/60 Hz. The combined period of vibration and spindle rotation in the meridional direction is

(28) $$T=\frac{\left[f_{V},f_{n}\right]}{f_{V}}T_{n}=\frac{60\left[f_{V},f_{n}\right]}{nf_{V}}$$

where [*f_v_*, *f_n_*] denotes the least common multiple.

Simulations were conducted using the vibration parameters in Table 5 (with *φ_i_* = 0), resulting in the surface topography shown in Figure 6b,d.

**Figure 8:**
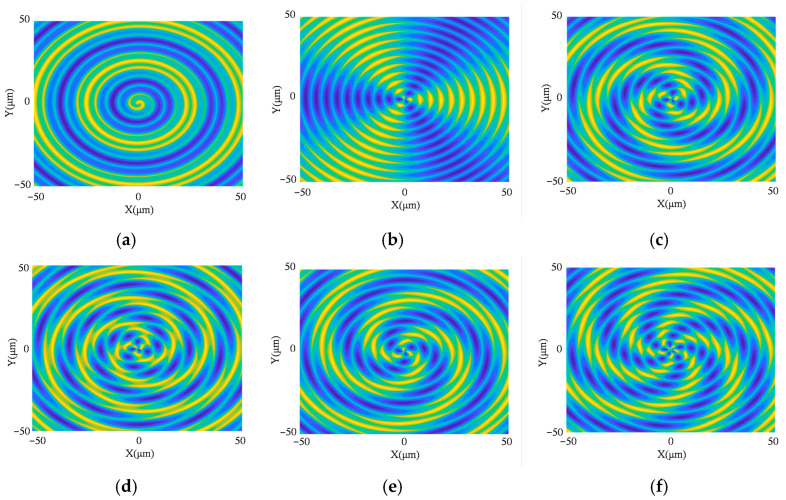
Turning surface patterns under different vibration frequency ratios: (**a**) I = 0, D = 0.2; (**b**) I = 3, D = 0; (**c**) I = 3, D = 0.2; (**d**) I = 3, D = 0.3; (**e**) I = 3, D = −0.2; (**f**) I = 5, D = −0.2.

The resulting surface pattern depends on the ratio between vibration frequency and spindle rotational frequency:

(29) $$\frac{f_{V}}{f_{n}}=I+D$$

where *I* is a non-negative integer, and *D* is a decimal within (−0.5, 0.5]. Simulations varying between *I* and *D* (Figure 8) show that *I* determines the number of pattern lobes (*I* = 0 yields concentric rings), while the sign of *D* governs the rotation direction, and |*D*| controls the spiral curvature. Straight patterns occur at *D* = 0.

**Figure 9:**
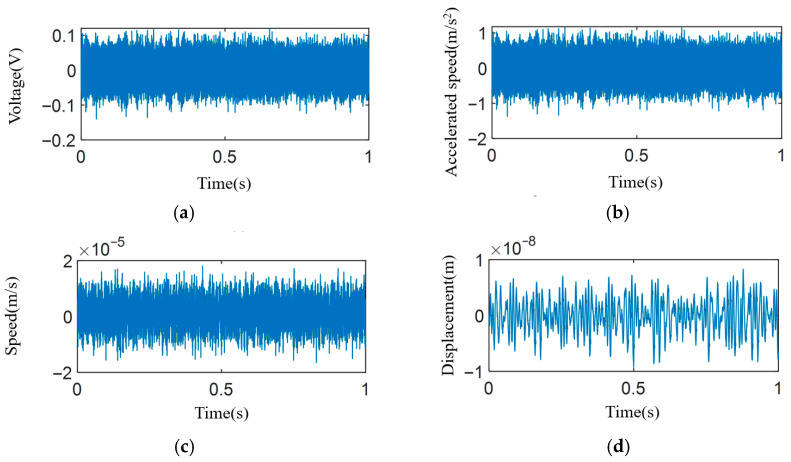

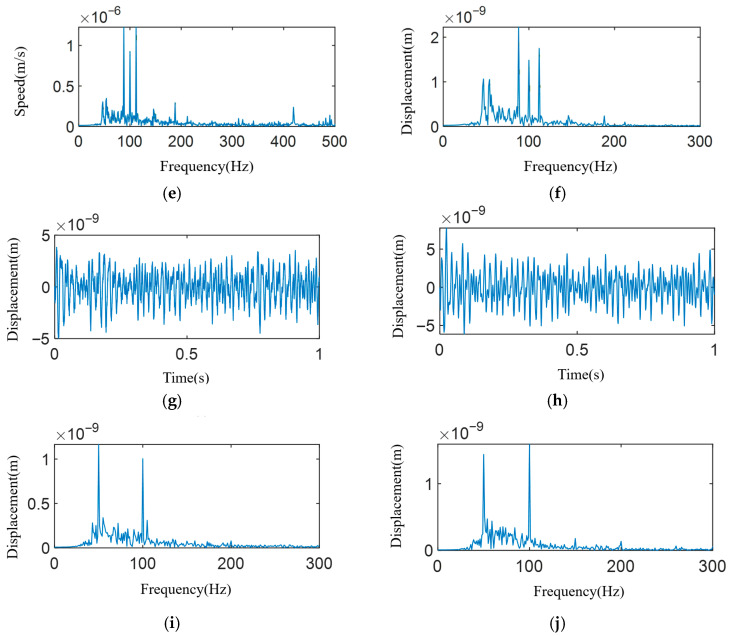
Measurement and analysis of *Z*-axis vibration during machining and under idle conditions. (**a**) Raw voltage–time signal acquired from the accelerometer. (**b**) Acceleration–time waveform converted from the voltage signal. (**c**) Velocity–time waveform obtained via integration. (**d**) Displacement–time waveform (final vibration displacement). (**e**) Frequency spectrum of the velocity signal. (**f**) Frequency spectrum of the displacement signal. (**g**) Displacement–time waveform with the machine powered off (background/environmental vibration). (**h**) Displacement–time waveform with the machine idling, but the tool disengaged from the workpiece. (**i**) Frequency spectrum corresponding to (**g**). (**j**) Frequency spectrum corresponding to (**h**).

Vibration during actual machining was measured using a high-precision Kistler accelerometer (±5 g range, 1000 mV/g sensitivity, 0–20 kHz frequency range). The acquired electrical signals were converted to displacement via a time–frequency hybrid integration method, which combines numerical integration in the time domain with spectral correction in the frequency domain to mitigate drift and noise [42]. The resulting *Z*-axis vibration signal is shown in Figure 9.

**Figure 10:**
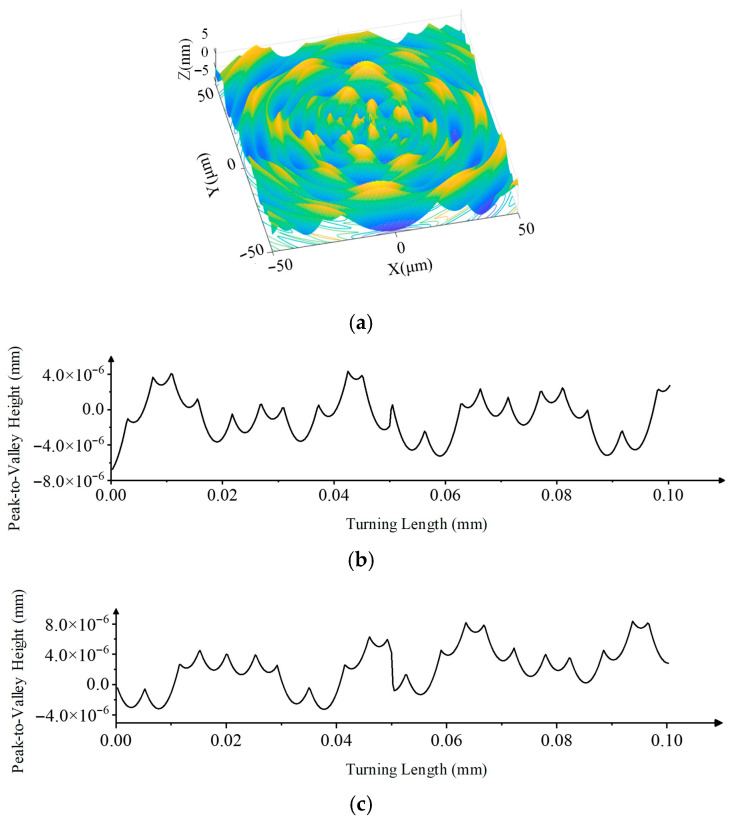
Analysis of profile errors from vibration simulations. (**a**) 3D simulation of ideal turning; (**b**) 2D profile of ideal turning; (**c**) 2D profile of turning with vibration.

Vibration signals with the machine off and idling (tool disengaged) were also measured (Figure 9g–j), revealing inherent environmental and machine frequencies at 50 Hz and 100 Hz. Subtracting these, the primary machining vibration components were identified (Table 6), and the corresponding simulated surface topography is shown in Figure 10.

A comparison of roughness (Ra) and waviness (Wz) under ideal, simulated vibration, and actual measured conditions is presented in Table 7.

**Figure 11:**
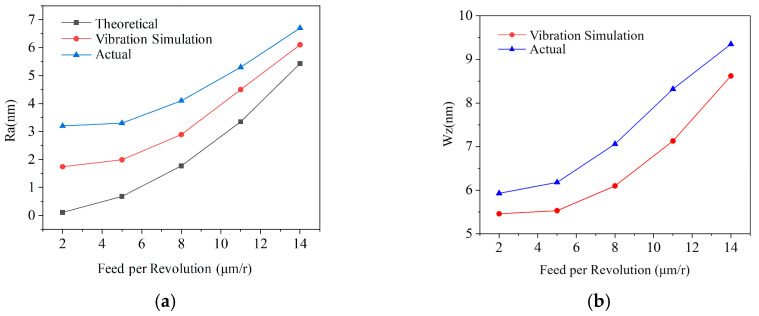
Influence of vibration on micro-topography: (**a**) roughness comparison; (**b**) waviness comparison.

Additional simulations and measurements were performed at feed rates of 0.01 mm/rev and 0.015 mm/rev. The trends in Ra and Wz (theoretical, simulated with vibration, and measured) are compared in Figure 11.

Under the given parameters, *Z*-axis vibration increased Ra by 301.5% compared to the ideal value, accounting for 61.2% of the actual measured Ra. Vibration-induced waviness constituted 89.5% of the total measured Wz. The data indicate that vibration significantly impacts roughness at low feeds, with its relative contribution decreasing as feed increases. Vibration is also the primary source of waviness.

### 3.4. Process Parameter Optimization

**Figure 12:**
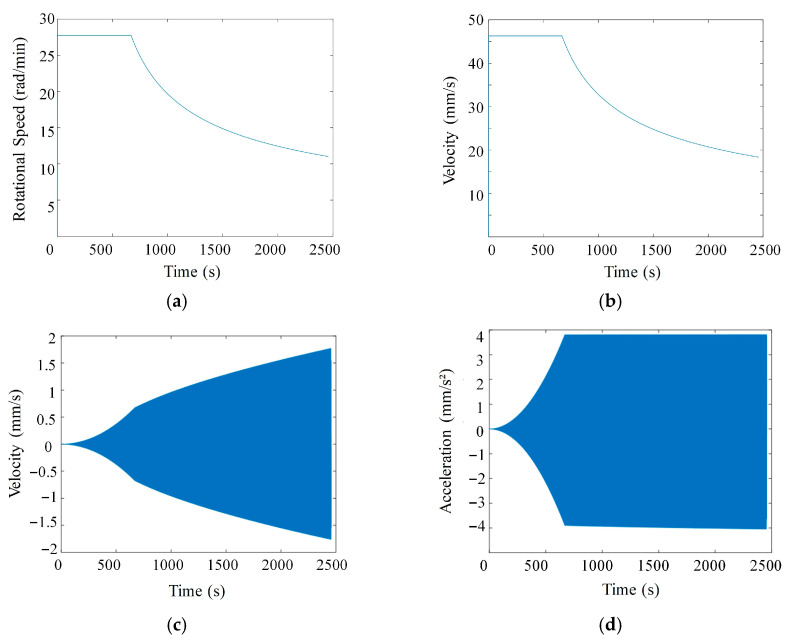
Motion profiles of machine axes: (**a**) Spindle speed trend; (**b**) *X*-axis velocity trend; (**c**) *Z*-axis velocity trend; (**d**) *Z*-axis acceleration trend.

In SPDT-STS machining, spindle speed (n) is a key parameter affecting efficiency. A hybrid trajectory strategy was employed, combining constant-angle discretization in the inner region with constant-arc-length discretization in the outer region. In the constant-angle zone, the spindle rotates at a constant speed. In the constant-arc-length zone, the spindle speed must adapt to curvature changes, exhibiting variable deceleration characteristics. Based on accuracy requirements, the angular resolution in the constant-angle zone was set to 0.5°, and the maximum cutting arc length was constrained to 0.27 mm. With a fixed time interval of 3 ms between discrete points, the *X*-axis feed rate varies linearly with the spindle speed, as shown in Figure 12a,b.

The *Z*-axis motion parameters (velocity and acceleration) are governed by the sagittal slope of the machining trajectory (Figure 12c,d), requiring the servo system to track surface curvature dynamically. Optimizing the coordinated control of the *X* and *Z* axes can improve efficiency and suppress contour error.

In cylindrical coordinates (*r*, *θ*, *z*), the following holds:

(30) $$\left\{\begin{array}{c} z=f\left(r,\theta \right)\\ F=nf \end{array}\right.$$

(31) $$\left\{\begin{array}{c} r=nft\\ \theta =\frac{2\pi n}{60}t \end{array}\right.$$

**Figure 13:**
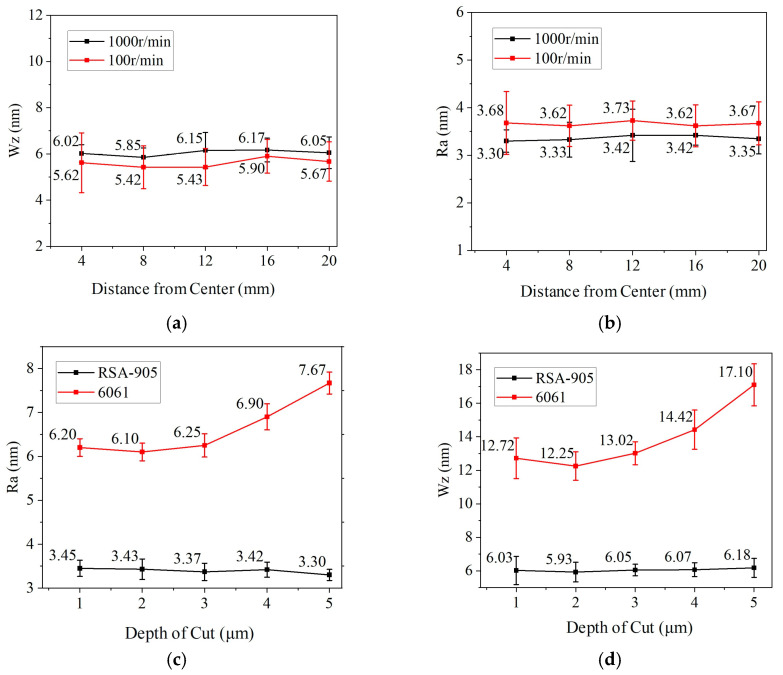

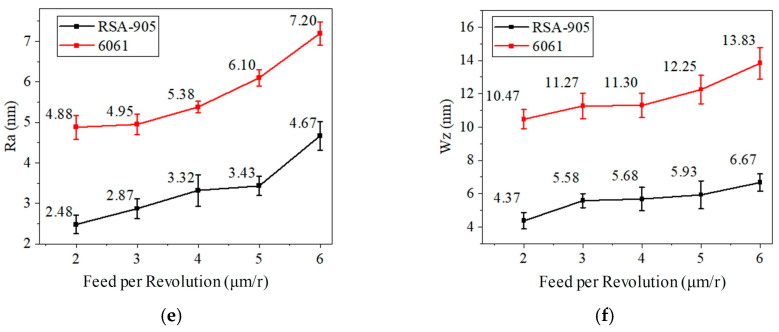
Surface error variation with process parameters: (**a**) Effect of speed on Ra. (**b**) Effect of speed on Wz. (**c**) Effect of depth of cut on Ra. (**d**) Effect of depth of cut on Wz. (**e**) Effect of feed per revolution on Ra. (**f**) Effect of feed per revolution on Wz.

Higher spindle speeds reduce machining time for a given feed. However, for non-rotationally symmetric freeform surfaces, the *Z*-axis undergoes reciprocating motion (Figure 13c,d), so the machine’s *Z*-axis dynamic capability must be considered. Dry-run tests of the trajectory program determined a minimum feasible time interval of 2 ms between points. Applying a safety factor of 1.5, a minimum interval of 3 ms was adopted.

Although spindle speed theoretically does not affect theoretical residual height (Ra), its practical influence was investigated. Plane turning experiments were conducted on Ø45 mm RSA-905 aluminum. Surface Ra and Wz were measured at radial intervals of 4 mm under two spindle speeds (100 rpm and 1000 rpm), with *f* = 5 μm/rev and depth of cut *h* = 3 μm. Results are in Table 8 and Figure 13a,b.

Results show marginally lower Ra at higher speed and slightly lower Wz at lower speed. Given the inherently low speeds in STS machining and assuming good chip evacuation, spindle speed has minimal impact on Ra and Wz. Therefore, higher speeds can be selected to improve efficiency without compromising accuracy.

Depth of cut (h) influences surface roughness primarily through residual stress and cutting force. Experiments on RSA-905 and 6061 aluminum were conducted at *n* = 1000 rpm, *f* = 5 μm/rev, with h varied from 1 to 5 μm in 1 μm steps. Results are in Table 9 and Figure 12c,d.

For 6061 aluminum, Ra and Wz increased with h. For microcrystalline RSA-905 (grain size 0.1–1 μm), surface quality remained stable across the tested range. The superior surface stability of RSA-905 compared to 6061 aluminum is likely attributable to its microcrystalline structure, which reduces localized plastic deformation and suppresses built-up edge formation during cutting, thereby enhancing surface integrity under varying depths of cut. Excessively small depths (≤1 μm) can lead to plowing rather than cutting, degrading surface quality. A depth of 2 μm is recommended, balancing surface quality, form accuracy, and tool life.

Feed per revolution (f) directly impacts surface quality and machining efficiency. Experiments on RSA-905 and 6061 aluminum were performed at *n* = 1000 rpm, *h* = 2 μm, with f varied from 2 to 6 μm/rev. Results are in Table 10 and Figure 13e,f.

Both Ra and Wz generally increased with feed, due to larger theoretical residual height and increased cutting forces/vibration. Feeds of 2–3 μm/rev yielded the best surface quality. A feed of 3 μm/rev is recommended to balance quality and efficiency.

## 4. Experiment and Discussion

### 4.1. Machining Equipment

**Figure 14:**
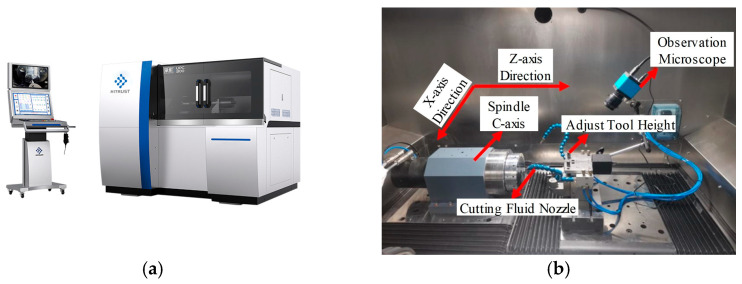
UPC-300 ultra-precision diamond turning lathe: (**a**) main structure; (**b**) inside view of the machine chamber.

The surface machining of the optical freeform mirror mold core was performed on a UPC-300 ultra-precision diamond turning lathe (Huacui Intelligent Equipment Co., Ltd., Taizhou, China). The machine structure is shown in Figure 14, with key specifications listed in Table 11.

The machine adopts a T-bed configuration with three axes. It is suitable for machining optical lenses, mirrors, mold cores, and precision mechanical components. Materials such as copper, aluminum, single-crystal germanium, zinc selenide, resin, and PMMA can be processed to optical quality finishes for flat, spherical, aspheric, off-axis, and freeform surfaces. The aperture of the HUD freeform mold core falls within the machine’s capacity. The machine can achieve nanometer-level form accuracy and sub-nanometer-level surface roughness, meeting the workpiece quality requirements.

A natural single-crystal diamond tool (Litian Century Diamond Tool Co., Ltd., Yuncheng, China) was selected for finishing. Its geometric parameters are detailed in Table 12.

### 4.2. Machining Experiment

Turning experiments on the HUD optical freeform mold core were conducted using the three-axis ultra-precision lathe and the natural single-crystal diamond tool. The workpiece material was microcrystalline aluminum alloy RSA-905 (Shanghai Weilian Industrial Co., Ltd., Shanghai, China). The surface was an XY polynomial freeform with dimensions of 138 mm × 68 mm. The machining process parameters for the final experiment are summarized in Table 13.

**Figure 15:**
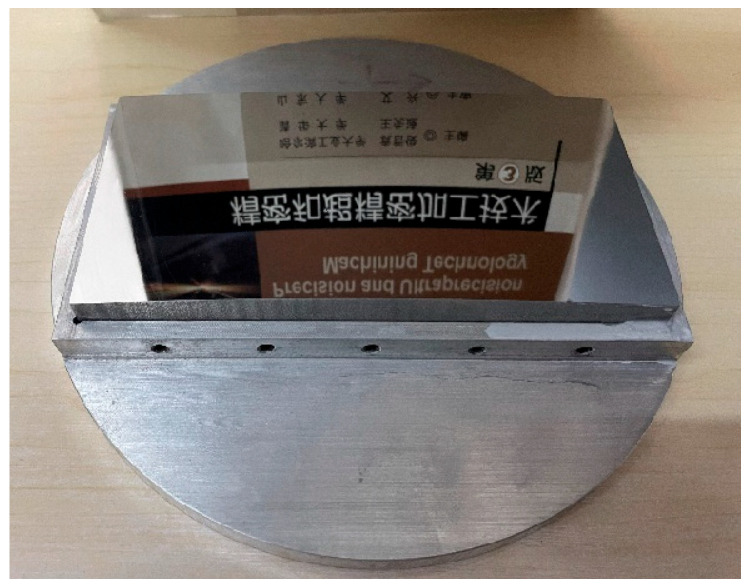
Photograph of the machined HUD optical freeform mold core.

The machined HUD freeform mold core is shown in Figure 15. The surface exhibited good specular reflection, with no observable machining marks, rainbow patterns, or other optical defects under light illumination.

### 4.3. Inspection and Discussion

**Figure 16:**
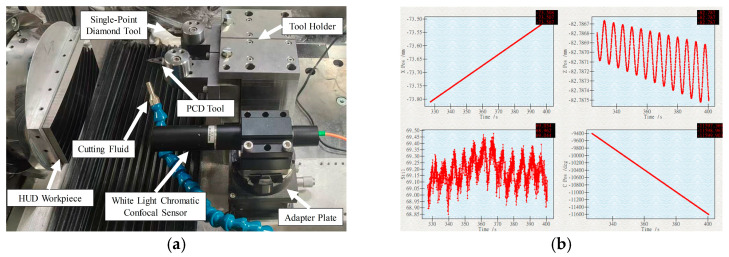
Form error inspection: (**a**) on-machine measurement setup; (**b**) data acquisition during on-machine measurement.

As noted in the process analysis, ultra-precision turning is time-intensive. If form error inspection after workpiece removal reveals that the target accuracy is not met, re-clamping introduces re-positioning errors and significantly increases the time cost of ma-chining and inspection cycles. To address this issue, after completing the turning process, the workpiece was not removed. Instead, the surface form error of the freeform surface was measured in situ using a scanning method (Figure 16a). If the measurement results did not meet the target, the error point cloud was used to compensate for the original freeform surface equation. The surface was then reconstructed, and the machining process was repeated until the inspection results met the target specifications before final work-piece removal.

An STIL CL1-MG140 chromatic confocal sensor was employed for on-machine meas-urement (Figure 16a). This sensor offers a measurement range of 150 μm, an accuracy of 20 nm, and a maximum measurable inclination of ±42.5°. To ensure all measurement points fell within the sensor’s range, the confocal sensor was mounted in place of the tool and moved along the original machining program. Data points were acquired every 10 ms (Figure 16b), enabling accurate surface reconstruction.

**Figure 17:**
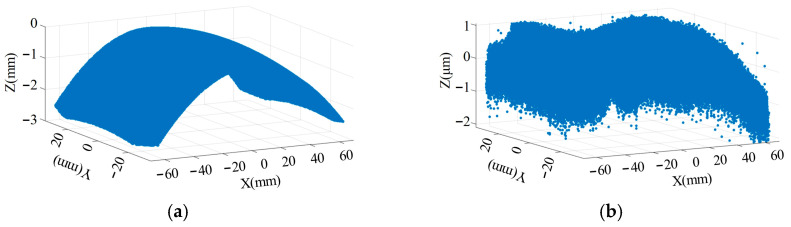
Form error from on-machine measurement: (**a**) reconstructed surface from fitted data; (**b**) contour map of form error.

The measured discrete points were used to reconstruct the surface and generate a form error contour map, as shown in Figure 17.

The calculated Peak-to-Valley (PV) form error was 3.1448 μm, and the Root-Mean-Square (RMS) error was 0.4085 μm. These values fell within the target range, meeting production requirements.

If the measured form error had exceeded the allowable limit, the error point cloud would have been refitted into an XY polynomial freeform surface. This error surface would then be used to compensate for the original optical freeform equation, generating a new machining trajectory. The ultra-precision machining process would be repeated iteratively until the measured form error was controlled within 5 μm. This approach establishes a closed-loop “machining–inspection–compensation” control system, effectively ensuring machining accuracy and efficiency.

**Figure 18:**
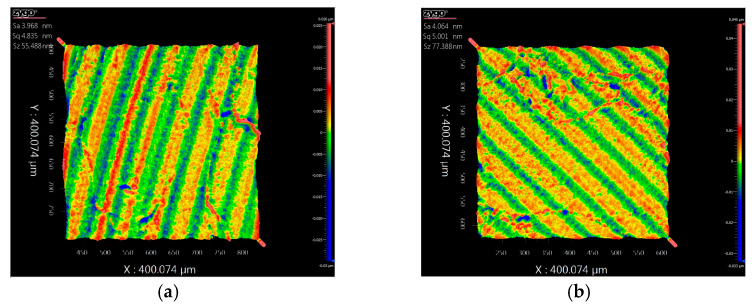
Surface roughness measurement results: (**a**) measurement location 1; (**b**) measurement location 2.

Surface roughness was measured using a Zygo white light interferometer with a 25× objective and a 400 μm × 400 μm field of view. The roughness measurement results are shown in Figure 18a,b. The achieved surface roughness (Ra) was approximately 4.02 nm, meeting production specifications.

**Figure 19:**
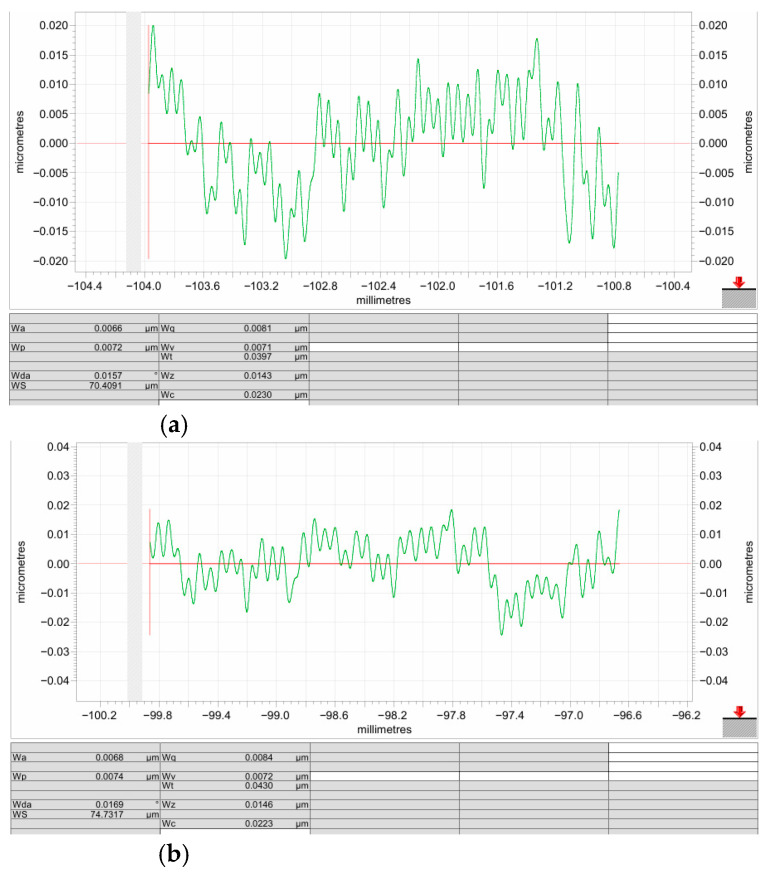
Surface waviness measurement results: (**a**) measurement location 1; (**b**) measurement location 2.

Surface waviness was measured using a Taylor Hobson profilometer with a 2 μm stylus tip radius and a 3.2 mm assessment length. The waviness measurement results are shown in Figure 19a,b. The waviness (Wz) was approximately 14.50 nm, which also met the production requirements.

## 5. Conclusions

This study presents a systematic optimization framework for ultra-precision machining of HUD freeform mold cores, with both practical and theoretical contributions to precision manufacturing science. This study systematically addressed the ultra-precision machining challenges associated with large-aperture, asymmetric freeform mold cores, focusing on optical design, tool path planning, process optimization, and vibration suppression. A comprehensive STS diamond turning process was developed, and its feasibility was experimentally validated. The main conclusions are as follows:

(1) A complete design and machining planning methodology for HUD freeform mold cores was established. An off-axis three-mirror optical system was modeled, and its surface parameters were optimized based on the XY polynomial freeform surface model. To accommodate the rectangular projection profile, a hybrid discretization strategy was proposed. This strategy combines constant-angle segmentation in the inner region with constant-arc-length segmentation in the outer region. Smooth and continuous tool paths were generated using cubic spline interpolation with specified second derivatives (three-bending-moment method), effectively ensuring both form accuracy and machining efficiency.
(2) The coupled influence mechanism of process parameters and machining vibration on surface quality was elucidated, and an optimized parameter set was determined. Machining vibrations along the *Z*-axis were identified as the primary cause of surface waviness. Through parametric optimization experiments, an optimal combination for machining microcrystalline aluminum RSA-905 was determined: a spindle speed of 1000 rpm, a depth of cut of 2 μm, and a feed per revolution of 3 μm/r. This parameter set yielded superior surface integrity, with good agreement between theoretical analysis and experimental results.
(3) A closed-loop manufacturing workflow integrating machining, *in situ* inspection, and compensation was implemented, successfully producing a qualified workpiece. Surface form was measured on-machine using a chromatic confocal sensor. The acquired data were used to compensate the design model iteratively through subsequent machining passes. The final freeform mold core achieved a peak-to-valley (PV) form error below 3.14 μm and a surface roughness (Ra) of approximately 4.02 nm, demonstrating the practical capability and stability of the proposed process.

This research provides a viable technical pathway for the independent ultra-precision manufacturing of core HUD optical components, contributing positively to the advancement of high-end optical manufacturing processes. While this study focused on microcrystalline aluminum, the proposed methodology is expected to be applicable to other common mold materials such as copper and nickel-plated steel, albeit with adjustments in tool geometry, cutting parameters, and vibration damping strategies to accommodate differences in material properties and machinability. Future work may focus on more complex surface types and optical materials, exploring areas such as multi-axis cooperative control and intelligent process optimization.

## Figures and Tables

**Table 1:** Surface parameters of the automobile front windshield.

Parameter	Value	Parameter	Value
*c*	5.9037 × 10^−5^	*k*	−1
*C*_1_	0.0317	*C*_2_	−9.9444 × 10^−10^
*C*_3_	1.9745 × 10^−9^	*C*_4_	−10.5564
*C*_5_	−6.1946 × 10^−11^	*C*_6_	−0.9550
*C*_7_	2.8881 × 10^−10^	*C*_8_	−5.2601 × 10^−10^
*C*_9_	8.2296 × 10^−10^	*C*_10_	−1.9323 × 10^−9^

**Table 2:** Virtual image parameters of the HUD system.

HUD System Parameter	Value
Field of View (FOV)	7° × 3°
Virtual Image Distance (VID)	2.5 m
Eyebox	130 mm × 50 mm
Down-angle	3°

**Table 3:** Specific parameters of the HUD optical freeform surface.

Parameter	Value	Parameter	Value
*c*	−4.4032 × 10^−4^	*k*	−1
*C*_1_	0	*C*_2_	0
*C*_3_	0	*C*_4_	−3.1027 × 10^−4^
*C*_5_	−2.9125 × 10^−5^	*C*_6_	−7.6812 × 10^−5^
*C*_7_	−2.4365 × 10^−8^	*C*_8_	2.5126 × 10^−7^
*C*_9_	−3.9835 × 10^−8^	*C*_10_	2.7781 × 10^−7^
*C*_11_	−2.5103 × 10^−10^	*C*_12_	−3.8860 × 10^−11^
*C*_13_	−4.9928 × 10^−10^	*C*_14_	1.5885 × 10^−10^
*C*_15_	−8.4241 × 10^−11^		

**Table 4:** Simulation parameters for ideal surface turning.

Process Parameter	Value
Tool Nose Radius, *R_t_*	1.2 mm
Spindle Speed, *n*	1000 rpm
Feed per Revolution, *f*	0.005 mm/rev
Cutting Depth, *h*	0.005 mm
Workpiece Size	0.1 mm × 0.1 mm

**Table 5:** Vibration parameters for surface turning simulation.

Vibration Component	Amplitude (nm)	Frequency (Hz)
1	1	90
2	2	105

**Table 6:** Main vibration signal parameters during turning.

Vibration Component	Amplitude (nm)	Frequency (Hz)
1	1.07	47
2	1.06	54
3	0.71	56
4	2.23	88
5	1.75	112

**Table 7:** Comparison of roughness and waviness results.

Evaluation Parameter	Theoretical Value	Vibration Simulation	Actual Measurement
Ra	0.67 nm	2.02 nm	3.30 nm
Wz	0 nm	5.53 nm	6.18 nm

**Table 8:** Effect of speed on roughness and waviness.

Radius, *r* (mm)	Speed, *n* (rpm)	Ra (nm)	*σ_Ra_* (nm)	Wz (nm)	*σ_Wz_* (nm)
4	100	3.68	0.66	5.62	1.29
	1000	3.30	0.24	6.02	0.38
8	100	3.62	0.44	5.42	0.93
	1000	3.33	0.37	5.85	0.41
12	100	3.73	0.41	5.43	0.80
	1000	3.42	0.54	6.15	0.78
16	100	3.62	0.44	5.90	0.73
	1000	3.42	0.20	6.17	0.51
20	100	3.67	0.45	5.67	0.85
	1000	3.35	0.32	6.05	0.68

**Table 9:** Effect of depth of cut on roughness and waviness.

Depth, *h* (μm)	Material	Ra (nm)	*σ_Ra_* (nm)	Wz (nm)	*σ_Wz_* (nm)
1	RSA-905	3.45	0.18	6.03	0.84
	6061	6.20	0.20	12.72	1.22
2	RSA-905	3.43	0.23	5.93	0.59
	6061	6.10	0.20	12.25	0.85
3	RSA-905	3.37	0.20	6.05	0.35
	6061	6.25	0.27	13.02	0.69
4	RSA-905	3.42	0.17	6.07	0.42
	6061	6.90	0.30	14.42	1.17
5	RSA-905	3.30	0.13	6.18	0.56
	6061	7.67	0.25	17.10	1.25

**Table 10:** Effect of feed per revolution on roughness and waviness.

Feed, f (μm/rev)	Material	Ra (nm)	*σ_Ra_* (nm)	Wz (nm)	*σ_Wz_* (nm)
2	RSA-905	2.48	0.22	4.37	0.49
	6061	4.88	0.29	10.47	0.57
3	RSA-905	2.87	0.24	5.58	0.46
	6061	4.95	0.26	11.27	0.77
4	RSA-905	3.32	0.39	5.68	0.71
	6061	5.38	0.15	11.30	0.73
5	RSA-905	3.43	0.23	5.93	0.84
	6061	6.10	0.20	12.25	0.85
6	RSA-905	4.67	0.35	6.67	0.52
	6061	7.20	0.29	13.83	0.95

**Table 11:** Main specifications of the machine tool.

System Configuration	Value
Maximum Machining Capacity	Ø300 mm, L300 mm
Spindle Rotation Accuracy	Axial runout ≤ 50 nm, Radial runout ≤ 50 nm
Maximum C-axis Speed	3000 rpm
Linear Axis (X/Z) Straightness	±0.2 μm/300 mm
Linear Axis (X/Z) Feed Rate	2.5 m/min

**Table 12:** Geometric parameters of the single-crystal diamond tool.

Diamond Tool Parameter	Value
Tool Nose Radius, *R_t_*	1127.46 μm
Effective Cutting Angle, *β*	120°
Rake Angle, *γ*	0°
Clearance Angle, *α*	10°
Waviness (Tool Edge)	0.043 μm

**Table 13:** Machining parameters for different stages in the HUD turning experiment.

Experimental Parameter	Semi-Precision Machining	Precision Machining	Ultra-Precision Machining
Depth of Cut, *h*	10 μm	3 μm	2 μm
Feed per Revolution, *f*	50 μm/rev	10 μm/rev	3 μm/rev
Max. Discrete Point Interval	270 μm	270 μm	270 μm
Sampling Points per Rev. (Constant-angle zone)	720	720	720
Time Interval Between Adjacent Points	3 ms	3 ms	3 ms

## Data Availability

The original contributions presented in this study are included in the article. Further inquiries can be directed to the corresponding author.
